# A subterminal growth zone at arm tip likely underlies life-long indeterminate growth in brittle stars

**DOI:** 10.1186/s12983-022-00461-0

**Published:** 2022-04-12

**Authors:** Vladimir Mashanov, Lauren Whaley, Kenneth Davis, Thomas Heinzeller, Denis Jacob Machado, Robert W. Reid, Janice Kofsky, Daniel Janies

**Affiliations:** 1 Wake Forest Institute for Regenerative Medicine, Winston Salem, NC USA; 2 Department of Biology, University of North Florida, Jacksonville, FL USA; 3 Edward Waters University, Jacksonville, FL USA; 4 Ludwig-Maximilian University, Munich, Germany; 5 University of North Carolina at Charlotte, Charlotte, NC USA

**Keywords:** Indeterminate growth, Echinodermata, Growth zone, Radial glia

## Abstract

**Background:**

Echinoderms are a phylum of marine invertebrates with close phylogenetic relationships to chordates. Many members of the phylum Echinodermata are capable of extensive post-traumatic regeneration and life-long indeterminate growth. Different from regeneration, the life-long elongation of the main body axis in adult echinoderms has received little attention. The anatomical location and the nature of the dividing progenitor cells contributing to adults’ growth is unknown.

**Results:**

We show that the proliferating cells that drive the life-long growth of adult brittle star arms are mostly localized to the subterminal (second from the tip) arm segment. Each of the major anatomical structures contains dividing progenitors. These structures include: the radial nerve, water-vascular canal, and arm coelomic wall. Some of those proliferating progenitor cells are capable of multiple rounds of cell division. Within the nervous system, the progenitor cells were identified as a subset of radial glial cells that do not express Brn1/2/4, a transcription factor with a conserved role in the neuronal fate specification. In addition to characterizing the growth zone and the nature of the precursor cells, we provide a description of the microanatomy of the four distal-most arm segments contrasting the distal with the proximal segments, which are more mature.

**Conclusions:**

The growth of the adult brittle star arms occurs via proliferation of progenitor cells in the distal segments, which are most abundant in the second segment from the tip. At least some of the progenitors are capable of multiple rounds of cell division. Within the nervous system the dividing cells were identified as Brn1/2/4-negative radial glial cells.

**Supplementary Information:**

The online version contains supplementary material available at 10.1186/s12983-022-00461-0.

## Background

Echinoderms are a phylum of marine invertebrates that constitutes a sister group to chordates within the monophyletic clade Deuterostomia. Echinoderms exhibit a range of phenomena that are of high fundamental interest to the fields of developmental biology and regenerative medicine. These phenomena include embryonic development [1, 2] and post-traumatic tissue and organ regeneration [3–6]. Among the least studied capacities of echinoderms is the life-long indeterminate adult growth [7, 8]. Indeterminate growth is considered an ancestral condition in animal phyla and correlates with the ability to regenerate damaged or lost body parts [9]. Therefore, it is important to understand indeterminate growth better to gain insights into the mechanisms of post-traumatic tissue regrowth and longevity.

Stellate echinoderms, such as starfish and brittle stars, are characterized by segmented body extensions called arms that radiate outward from the body’s center. The arms increase in length throughout the post-embryonic life of the animal. Based on the analysis of the skeletal plate arrangement in sea stars, Hotchkiss [7, 8] proposed a distalization-followed-by-intercalation model of adult arm growth. According to this model, the most distal part of the arm (arm tip) develops very early in the post-embryonic development. The distal growth of the arm is then sustained throughout the life of the animal by the development of new segments, not at the very tip, but in the zone just proximal to it [7, 8]. This hypothesis has never been confirmed in starfish or any other class of echinoderms. The cell sources and mechanisms of this axial growth also remain unknown.

Brittle stars are emerging model organisms in regenerative biology. They are capable of quickly regrowing their arms following autotomy. The molecular underpinnings of regeneration in these animals are being actively uncovered through functional [10], cellular [11], and genomic studies [12]. Here, we focus on the growth of the intact (i.e., non-regenerating) adult arms. Using a cell proliferation assay, we identify the growth zone in the arm’s second (sub-terminal) segment. We demonstrate that growth involves repeated cell divisions of at least some of the progenitor cells. Each major anatomical structure in the new arm segments arises from its own pool of progenitor cells in the growth zone. We identified these progenitors in the central nervous system as a subpopulation of radial glial cells that do not express the neurogenic transcription factor Brn1/2/4.

## Results

### Organization of the arm tip

Although the organization of the proximal brittle star arm segments has been studied in detail [13–15], the microanatomy of the arm tip in these animals has never been adequately documented in the literature. Since this background knowledge is critical for the understanding of the data on terminal growth presented below, we first provide a morphological description of the organization of the four terminal arm segments in adult non-regenerating individuals. The study was done on two brittle star species—*Ophioderma brevispinum* Say, 1825 and *Amphipholis kochii* Lütken, 1872. As described previously [15], no significant differences in the microanatomy of the proximal (mature) arm segments were observed between these two species. Likewise, the arm tips of both brittle stars show similar organization and, therefore, will be described together below.

**Fig. 1:**
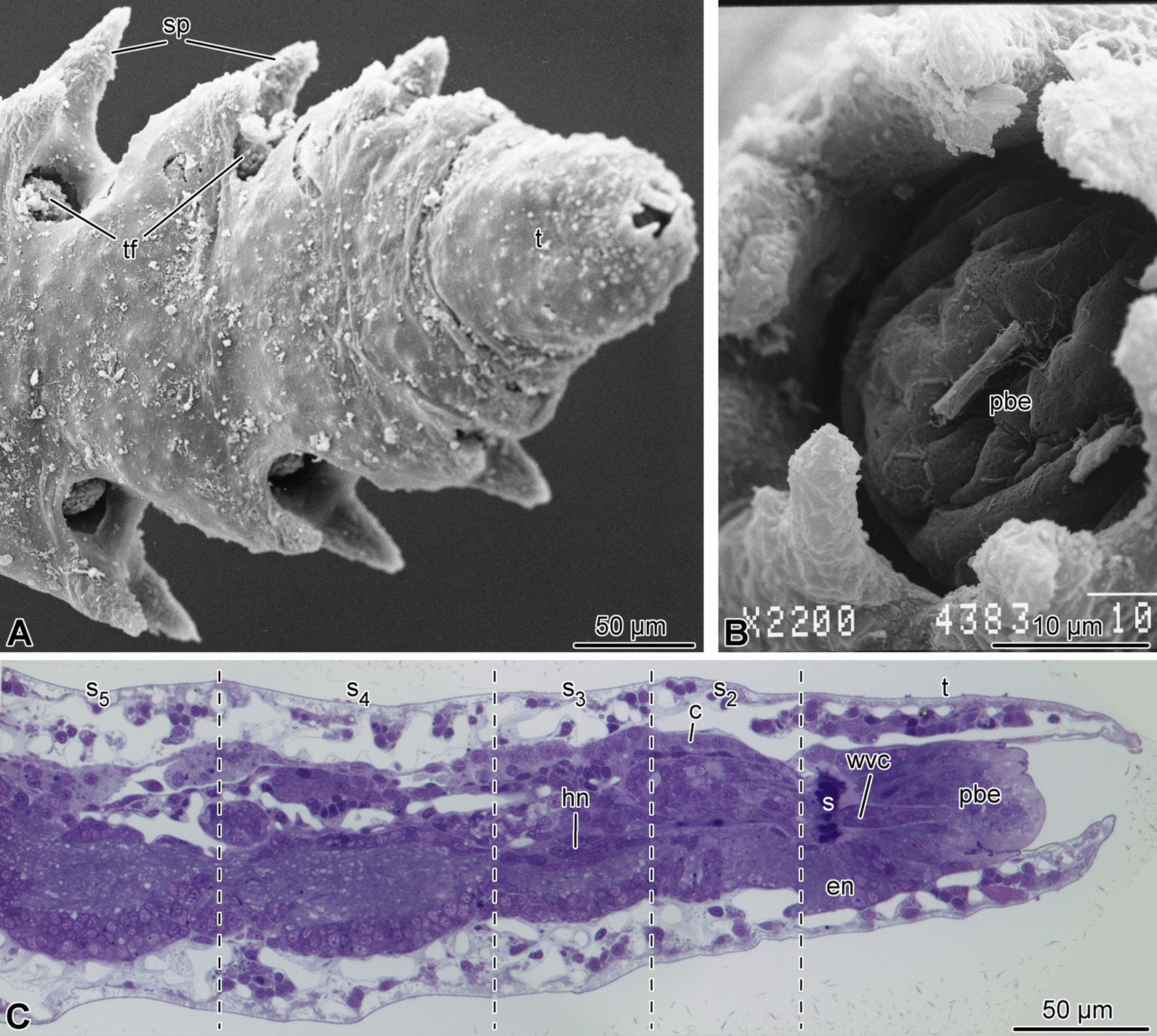
Overall morphology of the arm tip of *A. kochii*. **(a, b)** Scanning electron microscopy. **(a)** Low magnification view of the oral side. **(b)** Detailed view of the terminal pit. **(c)** Parasagittal semi-thin section. Toluidine blue. *c*—arm coelom; *en*—ectoneural neuroepithelium of the radial nerve cord; *hn*—hyponeural neuroepithelium of the radial nerve cord; *pbe*—epidermis of the terminal pit bottom; *s*—sphincter in the radial water-vascular canal; *s*$_2$–*s*$_5$—subterminal arm segments 2 thru 5; *sp*—arm spines; *t*—terminal segment; *tf*—tube foot; *wvc*—radial water-vascular canal

**Fig. 2:**
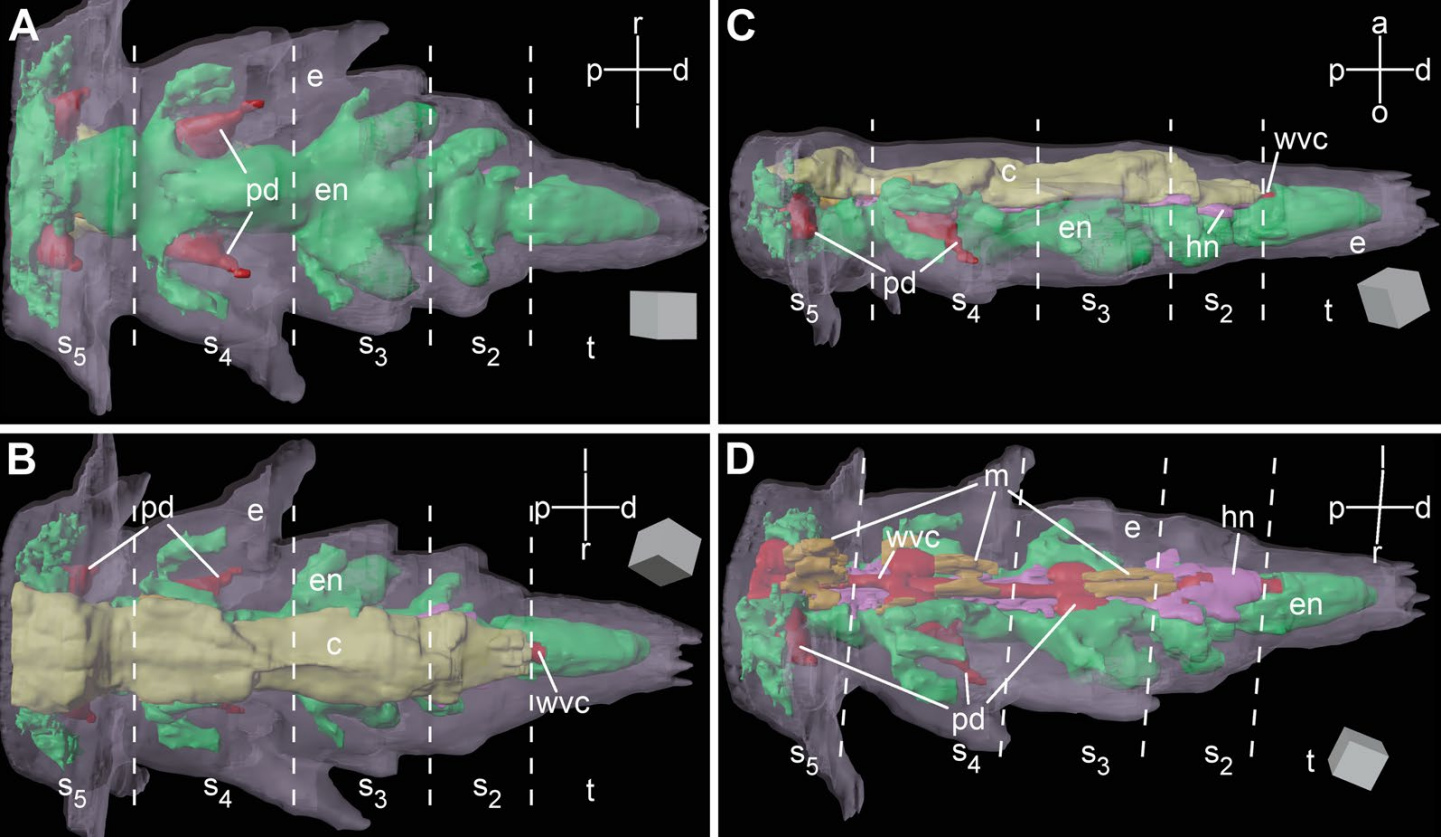
Three-dimensional reconstruction of the arm tip of *A. kochii*. The following anatomical structures are shown: epidermis *(semitransparent violet)*, ectoneural part of the nervous system *(green)*, hyponeural part of the nervous system *(magenta)*, water-vascular system *(red)*, arm coelom *(yellow)*, and intervertebral muscles *(brown)*. **(a)** Oral view. **(b)** Aboral view. **(c)** Side view. **(d)** Oblique side view, arm coelom not shown. The edge length of the scale cube *(grey)* is 25 $\mu$m. *a*—aboral; *c*—arm coelom (somatocoel); *d*—distal; *e*—epidermis; *en*—ectoneural system; *hn*—hyponeural system; *l*—left; *m*—intervertebral muscles; *o*—oral; *p*—proximal; *pd*—hydrocoelic lining of the podia (tube feet); *r*—right; *s*$_2$—*s*$_5$—subterminal arm segments 2 thru 5; *t*—terminal segment; *wvc*—hydrocoelic water-vascular canal. The 3D video animation of this anatomical model is available in Additional file 2. The original Blender model used to produce this figure and the video is available in Zenodo (https://doi.org/10.5281/zenodo.5762494)

**Fig. 3:**
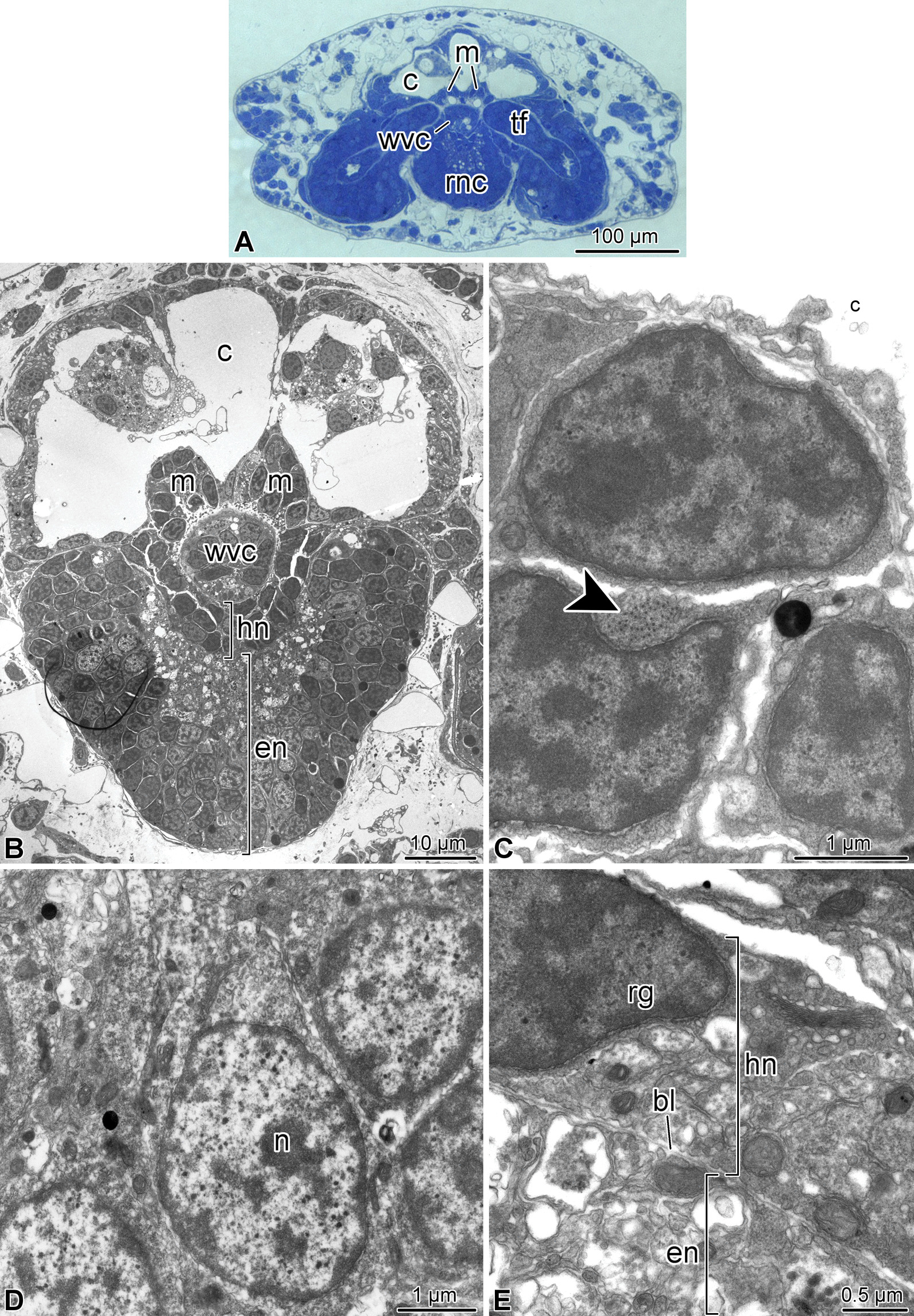
Organization of the fourth arm segment of *A. kochii*. Cross sections. **(a)** Low magnification view. Toluidine blue. **(b–e)** Transmission electron microscopy. **(b)** Low magnification transmission electron micrograph of the radial nerve cord, water-vascular canal, arm coelom, and intervertebral muscles. **(c)** High magnification view of the intervertebral muscle. *Arrowhead* shows the cytoskeletal filaments of the contractile apparatus. **(d)** Neuronal perikaryon in the ectoneural neuroepithelium. **(e)** Hyponeural part of the radial nerve cord. *bl*—basal lamina separating the ectoneural and hyponeural neuroepithelia; *c*—arm coelom; *en*—ectoneural neuroepithelium of the radial nerve cord; *hn*—hyponeural neuroepithelium of the radial nerve cord; *m*—intervertebral muscle; *n*—neuronal perikaryon; *rg*—radial glial cell; *rnc*—radial nerve cord; *tf*—tube foot; *wvc*—water-vascular canal

Brittle star arms are long segmented body appendages. Arm segments become progressively smaller towards the distal end (Figs. 1, 2). The overall organization of the third and fourth terminal segments (counting from the tip) is similar to the anatomy of more proximal arm segments, which has been extensively described elsewhere [13–15]. Briefly, the radial nerve cord (RNC) runs along the oral side of the arm (Figs. 1c, 2, 3a, b), beneath the oral skeletal shield and is composed of the thicker ectoneural and thinner hyponeural components (Figs. 1c, 2d, 3b, d, e). The radial water-vascular canal overlays the aboral side of the hyponeural cord and gives off two podia in each segment (Fig. 2). The arm coelom occupies the aboral side of the arm and covers the paired intervertebral muscles (Figs. 1c, 2b–d, 3a, b). However, there are also a number of differences in organization between these sub-terminal segments and more proximal segments of the arm: (a) there is only one pair of intervertebral muscles instead of two (i.e., oral and aboral) (Figs. 2d, 3a, b), these muscles are very small (Fig. 3a, b) and the contractile apparatus in the myocytes is weakly developed (Fig. 3c); (b) the arm coelom is expanded instead of being flattened (Fig. 3a, b), (c) even though the ectoneural part of the RNC contains prominent neuronal cells bodies and extensive neuropil, the hyponeural part is formed mostly by flattened glial cells surrounding scattered bundles of neuronal processes and very few neuronal perikarya (Fig. 3b, d, e).

**Fig. 4:**
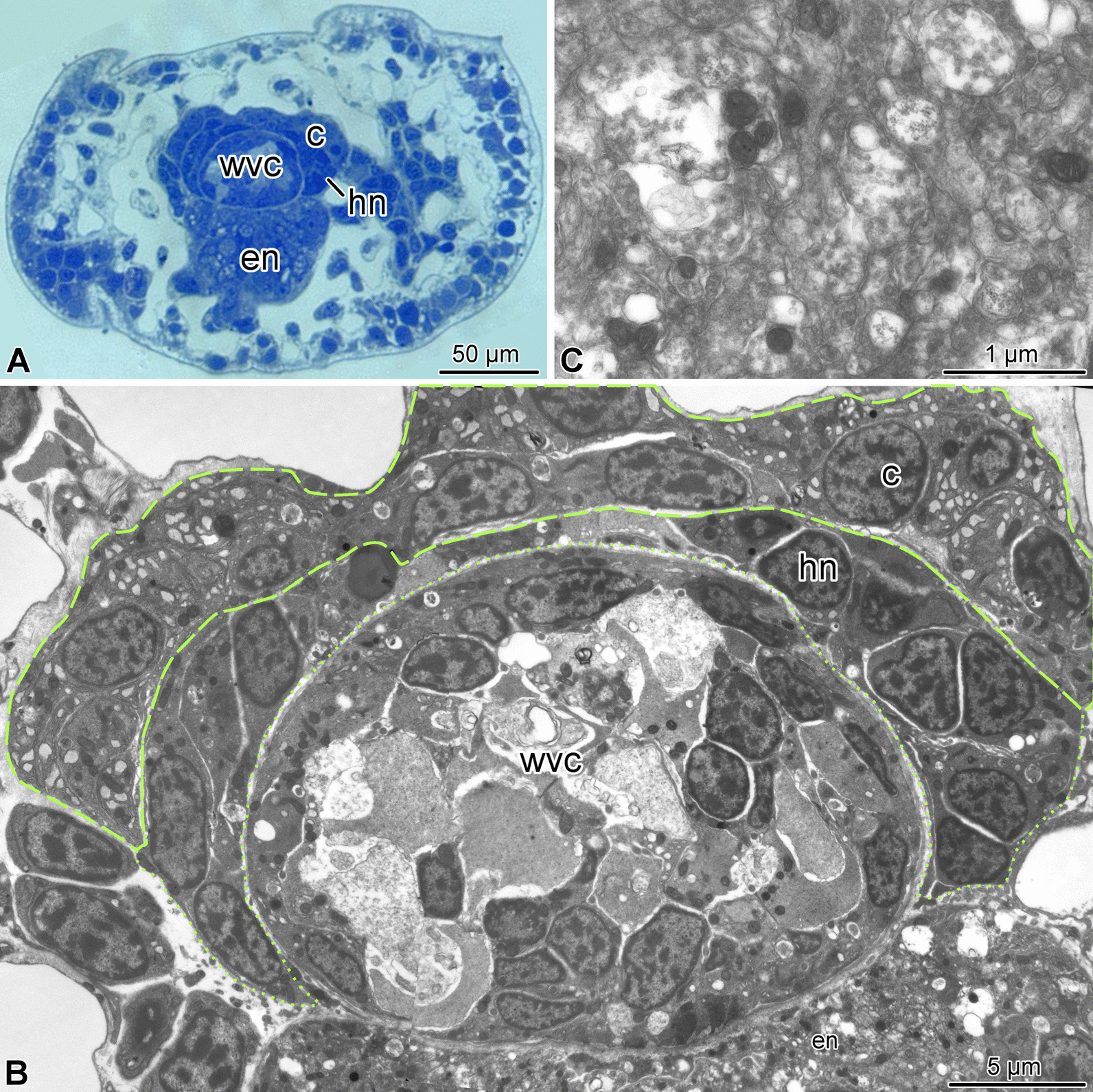
Organization of the second arm segment of *A. kochii*. Cross sections. **(a)** Low magnification view. Toluidine blue. **(b, c)** Transmission electron microscopy. **(b)** Overview of the radial nerve cord, water-vascular canal, and arm coelom. **(c)** High magnification view of the ectoneural neuropil. *c*—arm coelom; *en*—ectoneural part of the radial nerve cord; *hn*—hyponeural part of the radial nerve cord; *wvc*—water-vascular canal. *Dashed lines* indicate the arm coelom; *dotted lines* show the boundaries of the hyponeural part of the radial nerve cord

The two most terminal segments of the arm are markedly different in their organization from the proximal segments. In the second (sub-terminal) segment, the hyponeural cord bifurcates and the two branches ascend aborally along either side of the water-vascular canal and then fuse again in the space between the arm coelom and the water-vascular canal (Figs. 2d, 4a, b, 7a). This terminal hyponeural loop gives off a number of short narrow tracts that extend into the terminal segment (Fig. 7a). Intervertebral muscles are absent in this segment (Fig. 2d). At the tissue level, the neuroepithelium of the ectoneural part of the radial nerve cord still contains neuronal cell bodies and extensive neuropil (Fig. 4c), while the hyponeural cord is made up of mostly glial cells (Fig. 4b).

**Fig. 5:**
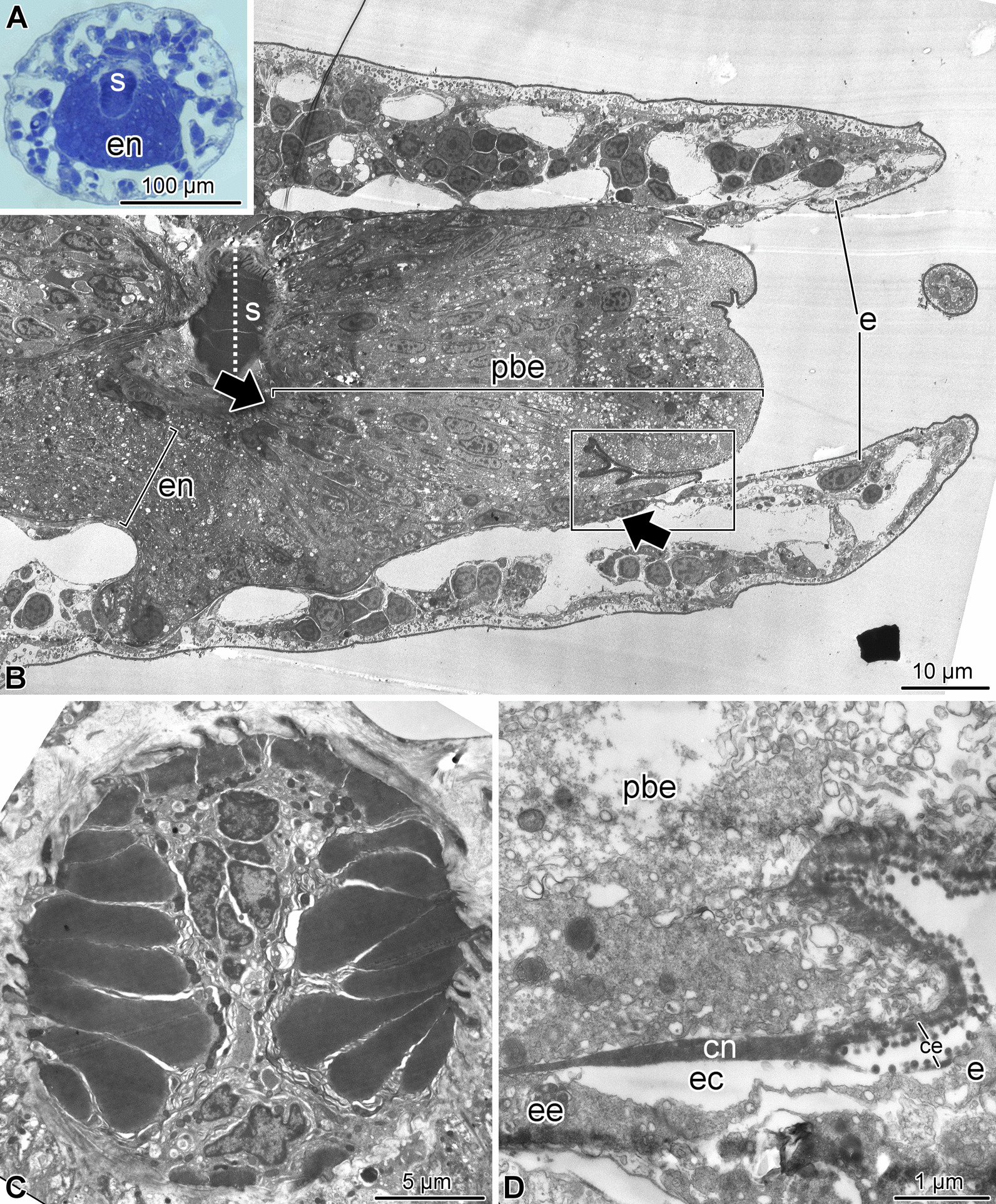
Overall organization of the terminal arm segment of *A. kochii*. **(a)** Low magnification view of a cross section. Toluidine blue. **(b–d)** Transmission electron microscopy. **(b)** Parasagittal section. Two opposing *arrows* indicate the level where the fusion between the ectoneural neuroepithelium and the epidermis takes place. **(c)** Cross section through the sphincter in the water-vascular canal at a level indicated by a dotted line in **(b)**. **(d)** Detailed view of a parasagittal section showing the fusion between the epineural cuticle *(cn)* and the cuticle of the surface epidermis *(ce)*. It approximately corresponds to the boxed area in **(A)**. *ce*—cuticle of the epidermis; *cn*—epineural cuticle; *e*—surface epidermis; *ec*—epineural canal; *ee*—epineural epithelium; *en*—ectoneural neuroepithelium of the radial nerve cord; *pbe*—epidermis of the pit bottom; *s*—sphincter in the water-vascular canal

**Fig. 6:**
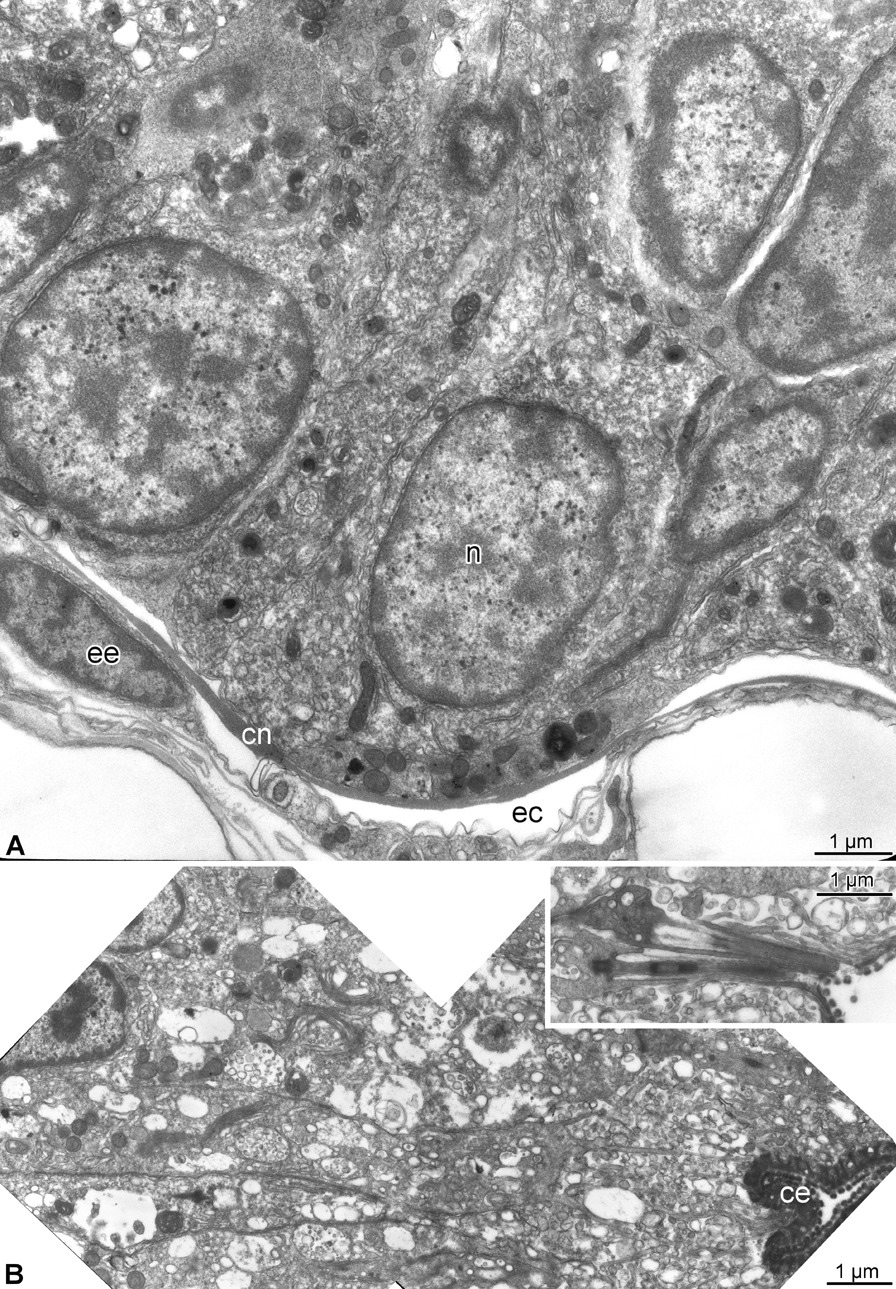
Ectoneural epithelium and terminal pit epidermis in the terminal arm segment of *A. kochii*. Transmission electron microscopy. **(a)** Cross-section of the ectoneural neuroepithelium of the radial nerve cord before its fusion with the epidermis of the terminal pit. **(b)** Sagittal section through the epidermis of the terminal pit. *Inset* shows the cilium and microvilli on the apical surface of the pit epidermis. *ce*—epidermal cuticle; *cn*—epineural cuticle; *ec*—epineural canal; *ee*—epineural epithelium; *n*—neuronal perikaryon

The terminal, distal-most, segment has a unique organization and deviates in its anatomy even further from all other arm segments (Figs. 1, 2,  5,  6). This terminal segment bears no spines and no tube feet. A single hollow tubular terminal ossicle forms the skeleton. The distal opening of this ossicle leads into a terminal pit. The bottom of the pit is lined with the epidermis composed of unusually narrow and tall ciliated epithelial cells (Figs. 1b, c, 5b, 6b), which are different from the epidermal cells elsewhere in the body [14]. Unlike the normally flattened epithelium that covers most of the animal, the terminal pit cells are filled with numerous vacuoles and have a well-developed Golgi complex (Fig. 6b). The terminal segment lacks the coelom and the hyponeural part of the radial nerve cord (Figs. 1c, 2b–d, 5a, b), as both structures terminate in the second sub-terminal segment. The only radial organs remaining are the ectoneural part of the RNC and the water-vascular canal (Figs. 2b–d, 5a–c). At the border between the terminal and the second sub-terminal segments, the water-vascular canal forms a sphincter composed of enlarged myoepithelial cells with a hypertrophic contractile apparatus (Figs. 1c, 5a–c). These powerful cells are mostly embedded into the lateral walls of the canal. At about the level of the sphincter in the water-vascular canal, the distal end of the ectoneural part of the RNC merges with the arm tip epidermis (Figs. 1c, 5b). The ectoneural neuroepithelium fuses with the region of the epidermis, which forms the bottom of the terminal pit, whereas the epineural epithelium is continuous with the flattened epidermis that covers the terminal skeletal ossicle (Fig. 5d). Topologically, the epineural canal opens into the outside environment. However, the opening is sealed by a T-junction formed by the fusion of the extracellular epineural cuticle with the cuticle that covers the apical surface of the epidermis (Fig. 5d).

**Fig. 7:**
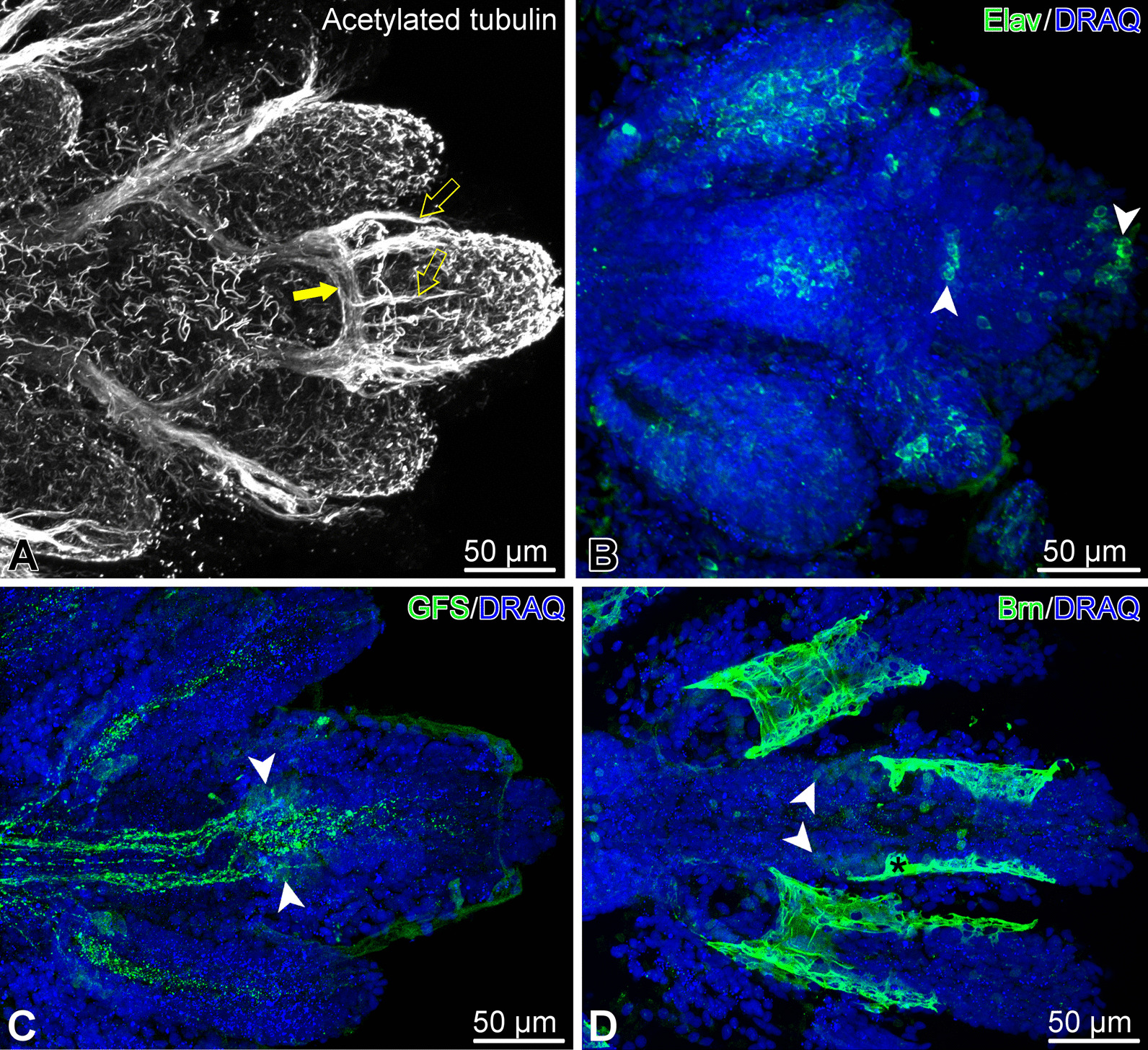
Organization of the nervous system in the arm tip of *O. brevispinum*. Maximum intensity Z-projections of confocal image stacks. **(A)** Acetylated tubulin. *Filled arrow* shows the terminal aboral loop formed by the hyponeural part of the radial nerve cord, which gives off a number of short tracts *(open arrows)* running towards the tip. **(B)** Elav-positive neurons. **(C)** GFSKLYFamide (GFS)-positive neuronal elements. **(D)** Expression of the transcription factor Brn1/2/4. Note that this particular antibody, besides specifically binding to the antigen in the nucleus, also non-specifically bind to the cuticle that covers the surface of the epidermis (*asterisk*)*White arrowheads* in **B–D** show clusters of immunopositive neuronal cell bodies

Before merging with the epidermis, the ectoneural neuroepithelium in the terminal segment still contains neuronal cell bodies and regions of neuropil (Fig. 6a). The distribution of different neuronal cell types in the terminal segment is however clearly different from that previously described for more proximal arm segments [15]. The antibody against the neuron-specific RNA-binding protein Elav labels the majority of the RNC neurons in the proximal arm segments [15]. In contrast, the Elav-positive neurons in the terminal segment are collected in a single prominent cluster at the very tip and also form a smaller cluster at the base of the segment (Fig. 7b).

In the RNC of the more proximal segments, the neurons containing the neuropeptide GFSKLYFamide were scattered throughout the ectoneural neuroepithelium and did not form clusters, while their processes were organized into distinct longitudinal tracts [15]. In contrast, in the arm tip, those longitudinal tracts end at the base of the terminal segment, where several GFSKLYFamide-immunopositive cell bodies form a distinct cluster (Fig. 7c).

Brn1/2/4 is a transcription factor of the POU family with an evolutionarily conserved role in neurogenesis [16–18]. It is abundantly expressed in the brittle star nervous system, including the terminal region of the RNC (Fig. 7d). We have previously shown that, in the proximal segments of the arm, Brn1/2/4 is expressed in all neurons in the ectoneural part of ganglionic swellings of the radial nerve cord. In addition to neurons, Brn1/2/4 is also present in a subset of radial glial cells [15]. In the arm tip, Brn1/2/4-postivie cells form a bilaterally symmetrical cluster at the base of the terminal segment (Fig. 7d), which is similar in size and position to that formed by GFSKLYFamide-positive cells. Some Brn1/2/4-positive cells are also scattered throughout the length of the terminal segment.

### Cell proliferation in the arm tip

**Fig. 8:**
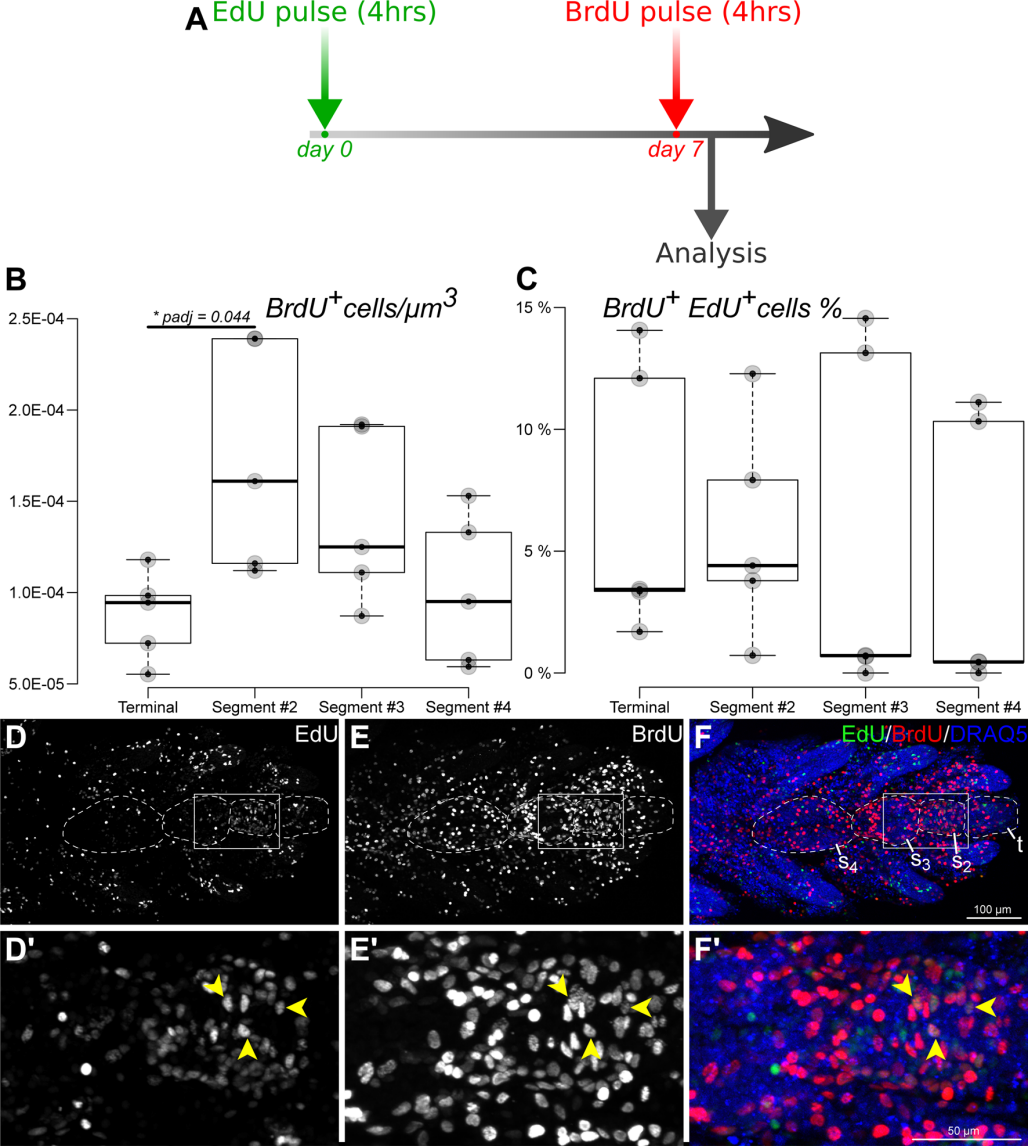
Quantification of cell proliferation in the four terminal arm segments *O. brevispinum*. **(a)** Dual pulse labeling paradigm. The animals were first pulsed with EdU, followed by a 7 day chase period, then pulsed with BrdU and processed for microscopic analysis. Both pulses lasted 4 h. **(b, c)** Boxplots showing individual data points and pairwise *P* values from the Wilcoxon-Mann-Whitney test. **(b)** Abundance of dividing (BrdU-incorporated) cells (per $\mu$m$^3$). **(c)** Relative abundance of repeatedly proliferating cells (i.e., those that have incorporated both thymidine analogs) measured as the percentage of proliferating cells undergoing the second round of cell division (i.e., the cells that have incorporated both EdU and BrdU). **(d–f’)** Representative micrographs showing distribution of labeled cells in the arm tip. Maximum intensity Z-projections of confocal stacks. Aboral-oral view. Boxed areas in **d, e**, and **f** indicated regions showed at higher magnification in **d’, e’, f’**, respectively. *Arrowheads* indicate dual-labeled EdU$^+$BrdU$^+$-cells *t*—terminal segment; *s*$_2$–*s*$_4$—arm segments 2 thru 4, as counted from the distal tip of the arm (outlined by dashed lines in **d’, e’, f’**)

**Fig. 9:**
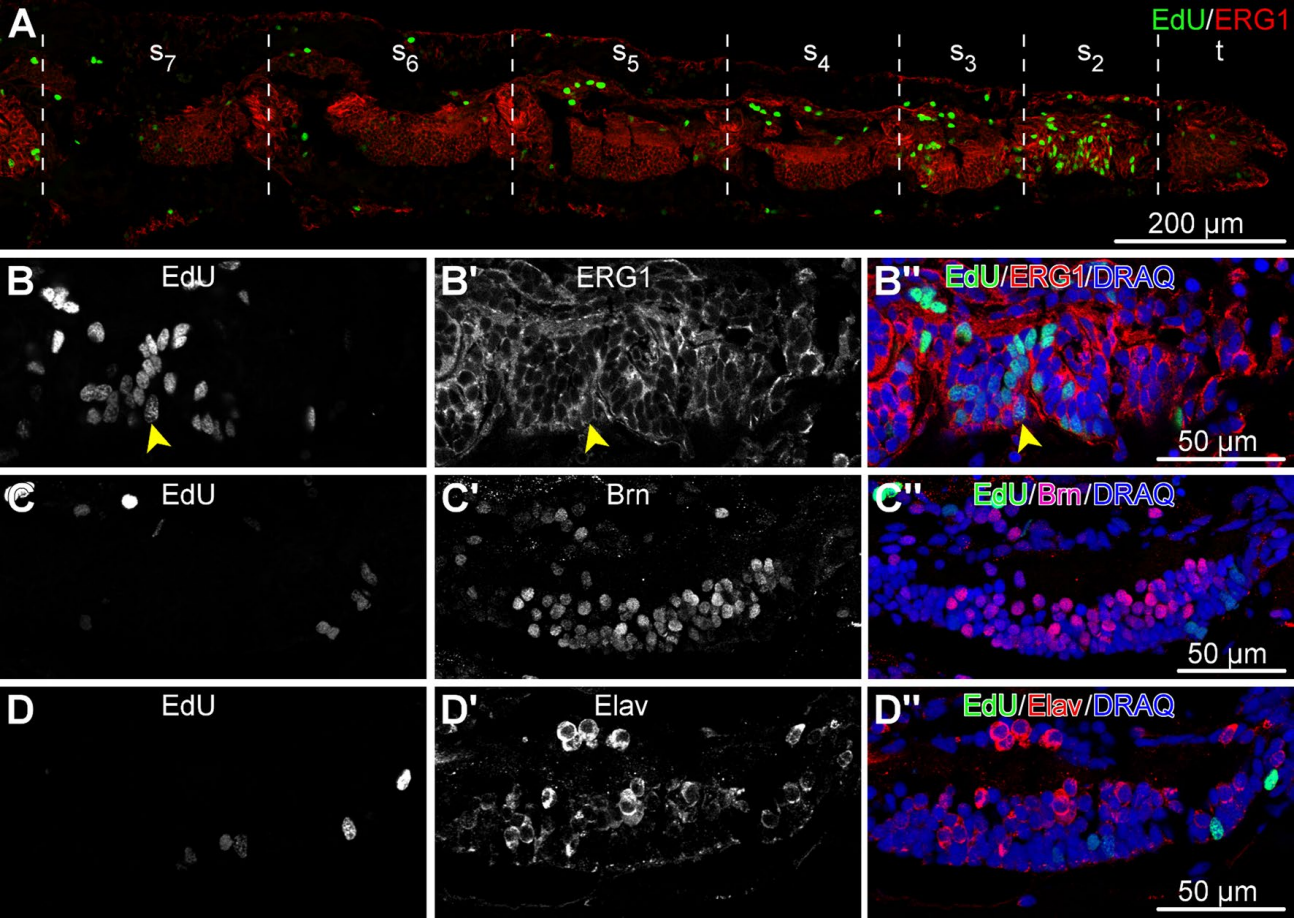
Distribution of dividing cells across the tissues and cell types in the arm tip segments *O. brevispinum*. The animals were pulsed with EdU for 4 h and processed for immunocytochemical staining. All micrographs are sagittal cryosections with the distal end to the right. **(a)** Low-magnification view of a sagittal section through the arm tip showing seven segments. **(b–d”)** Detailed views of the radial nerve cord in segment 2. **(b–b”)** Dual labeling with the EdU click reaction and ERG1 antibodies. The latter labels radial glial cells in the echinoderm central nervous system. *Arrowhead* points to a representative dual-labeled cell. **c–d”** Dual labeling with the EdU click reaction and neuronal markers Brn1/2/4 (**c–c”**) and Elav (**d–d”**). *t*—terminal segment; *s*$_2$–*s*$_7$—arm segments 2 thru 7, as counted from the distal tip of the arm

Cell proliferation in proximal segments of non-regenerating brittle star arms is negligible (Mashanov et al., in preparation). In order to establish if there is a distinct growth zone in the arm tip, we quantified the dividing cells in the four terminal arm segments. A snapshot of cell proliferation in the arm tip (i.e., the distribution and abundance of dividing cells at a given moment of time) was assessed by using a short (4 h) pulse of the thymidine analogs BrdU (Fig. 8a, b). Cell division occurred in all four distal-most segments, but the statistical analysis (Additional file 1) showed that the abundance of proliferating cells varied depending on the position of the segment relative to the tip (one-way ANOVA: *F* = 3.567 and *P* = 0.038). More extensive cell division occurred in the second (sub-terminal) segment from the arm tip compared to the tip (Figs. 8b, e–f’, 9a). On average, proliferating cells were twice as abundant in the second segment as in the terminal segment (Tukey’s test *P* = 0.044). Although not significantly different from segment 2, cell proliferation in the more proximal segments (the 3rd and 4th), gradually diminished. Within each of the four analyzed segments, the proliferating cells were seen in all major anatomical components of the arm, including the epidermis, arm coelom, and water-vascular system, but in the second segment they are mostly concentrated in the radial nerve cord (Fig. 9a).

We next conducted experiments to test whether the dividing cells were continuously proliferating (i.e., were capable of undergoing cell division more than once). To this end, we employed a dual pulse labeling when an additional pulse with the different thymidine analog, EdU, was administered 1 week prior to the BrdU pulse (Fig. 8a). This treatment showed that some (5.6–9.4%) of the previously divided cells can enter the mitotic cycle at least once again (Fig. 8c, d–f’). One-way ANOVA (*F* = 0.146, *P* = 0.931) shows no significant variation of the relative abundance of these repeatedly proliferating cells across the terminal segments. This suggests that in any segment with mitotic cells, the dividing cells are equally capable of undergoing repeated rounds of cell division.

To establish the identity of the dividing cells, we subjected the animals to a brief (4 h) pulse with EdU and then processed the tissue samples for cryosectioning and immunostaining with cell type-specific antibodies. In particular, we chose to focus our attention on the radial nerve cord in the second (sub-terminal) arm segment since this was the region where dividing cells were most abundant (Fig. 9). As a glial marker, we used the ERG1 antibody (Fig. 9a, b”), which labels radial glial cells in the echinoderm central nervous system [6, 15, 19, 20]. We also used an antibody against Brn1/2/4 (Fig. 9c–c”), a transcription factor that is expressed in all neurons and a subset of radial glia [15]. In addition, to label the neuronal lineage, we used the anti-Elav antibody (Fig. 9d–d”), which were raised against the respective protein from the sea urchin [18]. ELAV is a neuron-specific RNA-binding protein, which is expressed in the majority of neurons of the brittle star nervous system [15]. In the nervous system of the arm tip, all dividing cells are radial glia cells, as EdU labeling was exclusively co-localized with ERG1-positive cells but never with the cells that expressed Brn1/2/4 or Elav. This observation suggests that the neurogenic function at the arm tip is carried out exclusively by the Brn1/2/4-negative subset of radial glia.

## Discussion

In this study, we demonstrate that the proliferation of progenitor cells in the intact (i.e., non-regenerating) brittle star arms predominantly takes place in the second (counting from the arm tip) arm segment. This accumulation of dividing cells thus constitutes a subterminal growth zone that can enable the distal extension of the arm axis. Further studies are needed to dissect out the morphogenetic events that are downstream of the cell proliferation (i.e., the formation of the new segments per se versus their subsequent growth and extension).

We directly confirm an instance of the distalization-followed-by-intercalation model of adult echinoderm arm growth proposed by Hotchkiss [7, 8]. This conclusion is based on the statistical analysis of the distribution of dividing cells along the proximodistal axis of the arm. The omnibus one-way ANOVA test returns a significant result. Additional post-hoc Tukey’s test showed that the abundance of proliferating test was significantly higher in the second subterminal segment than in the terminal segment. However, given the sample size, we may question the assumption of parametricity. In response to that, we also performed the nonparametric Kruskal-Wallis test (Additional file 1). The Kruskal-Wallis test was marginally insignificant (*P* = 0.070), but the nonparametric Wilcoxon-Mann-Whitney test corroborated Tukey’s test results showing that, on average, proliferating cells were twice as abundant in the second segment as in the terminal segment (Wilcoxon’s test *P* = 0.036). Unlike in the terminal segment, the abundance of proliferating cells in segments 3 and 4 is not statistically different from that in segment 2. However, there still is a clear tendency of the median number of dividing cells to be higher in segment 2 than in segment 3, and in segment 3 versus segment 4. Taken together, these results indicate that the abundance of dividing cells is much higher in segment 2 than in the terminal segment. Cell proliferation in the more proximal segments (the 3rd and 4th) is gradually diminished compared to segment 2, but was also higher than in the terminal segment. This indicates that the 3rd and 4th segments (located proximal to the growth zone) undergo more extensive growth than the terminal segment (located distal to the growth zone).

Within the growth zone, the proliferating progenitors are found in all major tissues, including the radial nerve cord, water-vascular canal, arm coelom, and epidermis. We, therefore, hypothesize that the subterminal growth zone in the brittle star arm supplies cells to these respective components in the newly generated arm segments. Moreover, the data suggests that all these anatomical structures originate from the separate dividing progenitors in the growth zone and not from uncommitted pluripotent cells. This specialization of progenitor cells in the echinoderm growth zone is similar to what is observed in the chordate tail bud [21]. In the brittle star sub-terminal growth zone, we focused our attention on determining the nature of the proliferating cells within the central nervous system (the radial nerve cord). We established that only radial glia, not neurons, were capable of proliferation in the nervous system within the growth zone. This is in line with our previous finding that the radial glial cells function as progenitor cells giving rise to new glial and neuronal cells both in adult neurogenesis in intact animals [22] and in post-traumatic neural regeneration [6, 23].

Furthermore, we demonstrated that not all radial glial cells contribute equally to the extension of the radial nerve cord. Only the subset of the radial glia that did not express the transcription factor Brn1/2/4/could undergo cell division in the growth zone. Brn1/2/4 is a transcription factor of the POU family with a conserved role in neurogenesis and neuronal fate specification. Brn1/2/4 is understudied in echinoderm adults, but it has been shown to regulate the number of postmitotic neurons in the sea urchin larvae [18]. It has also been previously shown that in the brittle star central nervous system, this transcription factor is expressed in all neurons and also in a subset of radial glia [15]. However, the functional significance of the differences in gene expression between different subsets of radial glial cells has remained unknown. Here, we tie the absence of Brn1/2/4 expression to the ability of the radial glia to be involved in the adult distal growth of the radial nerve cords.

Analysis of the indeterminate growth in echinoderms provides additional insights into the evolution of the body plan in the phylum. Representatives of a wide range of bilaterian taxa can undergo indeterminate adult growth by adding new segments to their anterior-posterior axis. For example, adult annelids *Platynereis dumerilii* continuously produce a new body region via the action of the growth zone that is located in front of the pygidium (the posterior-most body part) [24]. Among chordates, amphioxus species have also been reported to add multiple segments to their trunk at the tailbud during post-embryonic life [21]. Overall, the elongation of the anterior-posterior axis by post-embryonic addition of new segments at the posterior sub-terminal zone was proposed to be an ancestral property of bilaterians [9, 24]. Even though the phylum Echinodermata belongs to the monophyletic clade Bilateria, adult individuals of all extant echinoderms have radial (often pentaradial symmetry), with multiple (often five) arms extending out from the center of the animal. The origin of this radially symmetrical body plan has been debated for decades. One of the hypothesis that has been recently getting traction postulates that early echinoderms (now extinct) were bilateral with a single main body axis. Later in evolution, a multiplication of the original axis took place through a yet unknown mechanism, with each new axis giving rise to an “arm”. Thus an adult radially symmetrical echinoderm can be viewed as a system of conjoined integrated “quintuplets”. A proximal-distal axis of each of the echinoderm arms thus can be homologous to the anterior-posterior axis of other bilaterians and the distal post-embryonic elongation of the echinoderm axes thus might be comparable to the posterior elongation of the anterior-posterior axis in other bilaterians. This is in line with the emerging consensus in the field that each of the five radii of adult echinoderms represents a separate axis of bilateral symmetry as a result of a multiplication of the original single axis in echinoderm evolution. This hypothesis has been supported by paleontological, molecular, and morphological studies [20, 25–27].

Another interesting aspect of the brittle star adult arm growth is its clear similarity to injury-induced arm regeneration. Shortly after autotomy or amputation, an outgrowth develops extending distally from the plane of the wound. Three distinct zones are then quickly established in this outgrowth: (1) a differentiated distal-most terminal segment, (2) a short subterminal growth zone, and (3) a continuously expanding differentiation zone. In the continuously expanding differentiation zone, the newly developing segments become progressively more differentiated with the increasing distance from the arm tip (i.e., in the distal-to-proximal direction) [11]. These parallels between post-traumatic regeneration and normal adult growth in the brittle star arm are in line with the observation that the capacity for indeterminate growth strongly correlates with the ability to regenerate body parts after injury. These results also support the idea that the mechanisms underlying the normal growth underlie post-traumatic regeneration [9]. Further comparative research of these two phenomena will undoubtedly contribute to better understanding of the evolution of the cellular and genetic underpinnings of growth and regeneration.

## Conclusions

- We provide a detailed description of the microanatomy of the brittle star arm tip and show that the two distal-most segments are markedly different from the proximal segments
- The growth of the adult non-regenerating arms is accomplished through the proliferation of the progenitor cells in the distal segments. These cells are most abundant in the second segment from the tip, forming a subterminal growth zone.
- Some of the proliferating progenitors are capable of repeated rounds of cell division.
- Each of the radial anatomical structures in the growth zone (the radial nerve cord, water-vascular canal, and arm coelom) has its own population of proliferating progenitor cells.
- In the nervous system (radial nerve cord) the dividing progenitor cells are identified as a Brn1/2/4-negative subpopulation of radial glia.

## Materials and methods

### Animals

Adult individuals of *Amphipholis kochii* Lütken, 1872 were collected from Vostok Bay, Sea of Japan (Russia). Individuals of *Ophioderma brevispinum* Say, 1825 were purchased from the Marine Biological Laboratory (Woods Hole, MA). The animals were maintained until needed in aerated filtered seawater at room temperature at a density of 1 animal per gallon. The study was performed on the animals that showed no signs of recent injury or regeneration (i.e., no scars or sharp differences in color or size along the arm).

### Electron microscopy

For transmission electron microscopy, the arm tips were fixed in 2.5% glutaraldehyde in cacodylate buffer (0.05M, pH 7.6, 1090 mOsm) overnight at 4 $^\circ$C, rinsed in the same buffer and postfixed in 1% OsO$_4$ for 1 h at room temperature. The calcareous endoskeleton was removed in 1% ascorbic acid prepared in 0.15M NaCl. The solution was changed daily until no skeletal elements could be observed with a stereo microscope. The specimens were then dehydraded in a graded ethanol series followed by acetone and embedded in the Araldite epoxy resin. Sections were cut with glass knives on a UC6 (Leica) ultramicrotome. Ultrathin (50–70 nm) sections were collected on Butvar (Sigma)-coated copper slot grids, stained with uranyl acetate and lead citrate, and then examined and imaged with a Zeiss EM 10 transmission electron microscope.

For scanning electron microscopy, the samples were fixed as above, dehydrated, critical point dried, and sputter coated with carbon and gold. They were then examined and photographed with a Jeol JSM-IC848 scanning electron microscope.

### 3D reconstruction

A complete series of transverse semithin (0.8 $\mu$m) sections was cut from one of the Araldite-embedded specimens. The sections were collected on gelatin-coated slides, stained with toluidine blue and then every third section was photographed with the Nikon Eclipse Ni microscope using a 40$\times$ objective. The image stack was imported into the TrakEM2 tool [28] within the Fiji/ImageJ software [29]. TrakEM2 was used for image registration, optical distortion correction, and initial 3D volume reconstruction. Final editing and rendering of the model was performed in the open source 3D modelling software Blender (https://www.blender.org). A 3D animation of the anatomical model is available in Additional file 2.

### Cell proliferation assay

To establish whether arm tip cells can undergo repeated rounds of cell division, we employed dual pulse labeling with two different thymidine analogs. First, individuals of *O. brevispinum* were incubated in a 5 $\mu$M EdU (5-Ethynyl-2’-deoxyuridine, Sigma Aldrich T511285) solution for 4 h in filtered seawater and then rinsed in three changes of clean seawater (1 h each). After a chase period of 7 days, the same animals were exposed to 50 mg/kg BrdU (5-Bromo-2’-deoxyuridine, Sigma Aldrich B5002) for 4 h and rinsed as above. After the second pulse, the arm tips were fixed at 4 $^\circ$C overnight in 4% paraformaldehyde prepared in 0.01M PBS (pH 7.4, 1020 mOsm). The tissue samples were then washed in PBS and decalcified in 10% EDTA in 0.05M Tris-HCl for 3 days. Next, the samples were bleached in increasing concentrations of H_2_O_2_ (0.3%, 1%, 3%, and 5%) and permeabilized by a mild Proteinase K treatment (2.5 $\mu$g/ml, 15 min at room temperature). The samples were incubated in 2M HCl for 30 min at 37 $^\circ$C to unmask the incorporated BrdU for subsequent immunocytochemical detection. EdU was detected first with copper-catalyzed click reaction between an alkyne on EdU and fluorescent Alexa Fluor 488 picolyl azide (Molecular Probes, C10637). The residual terminal alkyne groups on EdU were quenched in a click reaction with a non-fluorescent azide (20 mM azidomethylphenylsulfide, Sigma-Adlrich, 244546) [30] to prevent potential cross-reactivity of unreacted EdU with anti-BrdU antibodies. This treatment completely prevented the unwanted signal from appearing in our control samples. BrdU was detected with the rat anti-BrdU (GenWay: 20-783-71418) antibody, which was diluted 1:400 and applied for 48 h at 4 $^\circ$C. The unbound primary antibodies were removed by extensive washes in PBS (5 $\times$ 1 h). The secondary antibody (goat anti-rat Cy3, Jackson ImmunoResearch, 112-165-167) was also applied for 48 h at 4 $^\circ$C at the dilution of 1:2,000. Some of the cell proliferation assay samples were also subjected to immunostaining with the cell type-specific markers, as described below. The nuclei were stained with 5 $\mu$M DRAQ5 (Thermo Scientific), and the samples were mounted in an antifading medium containing 2.5% DABCO and 10% Mowiol 4-88 in 25% glycerol. Stacks of optical sections were taken with an Olympus FV1000 confocal laser scanning microscope. Images were processed and analyzed in the Fiji/ImageJ software [29].

Proliferating cells were counted in four terminal arm segments of five different individuals, using only one arm per individual. Maximum intensity Z-projections of confocal image stacks were used for quantification. Quantitative differences among the segments were analyzed with one-way ANOVA followed by Tukey’s post-hoc test in the R statistical environment [31]. However, considering the sample size, a nonparametric test could be more appropriate. Therefore, we also included a Kruskal-Wallis test followed by an ad hoc Wilcoxon-Mann-Whitney test.

### Immunocytochemistry

The arm tips of *O. brevispinum* were fixed in 4% paraformaldehyde and decalcified as above. Immunostaining was performed both on cryosections and whole mount tissue samples. For cryosectioning, the specimens were cryoprotected in sucrose and embedded in the Tissue-Tek OCT compound (Sakura). Cryosections (5–10 $\mu$m thick) were cut on a Leica CM1860 cryostat and collected on gelatin-coated slides. The slides were washed in PBS, incubated in 0.1M glycine for 1 h, and then blocked in 2% goat serum for 1 h. The following first antibodies were applied overnight at 4 $^\circ$C: mouse anti-acetylated tubulin (1:1,000, Sigma T6793), rat anti-Brn1/2/4 (1:1,000), rabbit anti-ELAV (1:3,000) [18], the echinoderm radial glial marker ERG1 (1:1, mouse) [19], and rabbit anti-GFSKLYFamide (1:1,000) [32]. The slides were then washed in PBS and the secondary antibodies [i.e., goat anti-mouse Cy3 (Jackson Immunoresearch, 115-165-146, 1:2,000), goat anti-rabbit FITC (ThermoFisher Scientific, 65-6111, 1:200), an goat anti-rat FITC (GenWay, GWB-7B4D70, 1:100)] were applied for 1 h at room temperature. The nuclei were stained with 5 $\mu$M DRAQ5 (Thermo Scientific) for 30 min and the slides were coverslipped in an anti-fading medium (2.5% DABCO, 10% Mowiol 4-88, 25% glycerol in 0.2M Tris-HCl, pH 8.5).

For whole-mount staining, the samples were fixed, bleached and permebealized with mild Proteinase K treatment as above. All wash buffers contained 0.5% Triton X-100. The incubation time in both the first and second antibodies was increased to 48 h.

Unless indicated otherwise, the micrographs are oriented with the oral side to the bottom and the distal end of the arm to the right.

## Supplementary information

**Additional file 1**. R Markdown file (R version 4.0.4). This file contains the data and statistical analysis about proliferating cells counted in the four terminal arm segments of five different individuals, using only one arm per individual

**Additional file 2**. 3D animation of the anatomical model of the *A. kochii* arm tip used in the creation of Fig. 2. The following anatomical structures are shown: epidermis (semitransparent violet), ectoneural part of the nervous system (green), hyponeural part of the nervous system (magenta), water-vascular system (red), arm coelom (yellow), and intervertebral muscles (brown).

## Data Availability

Supplementary information is available at Zenodo (https://zenodo.org/), DOI:https://doi.org/10.5281/zenodo.5762494. These materials include the high resolution video and Blender file used in the creation of Fig. 2.

## Abbreviations

BrdU: 5-Bromo-2’-deoxyuridine
EdU: 5-Ethynyl-2’-deoxyuridine
RNC: Radial nerve cord
